# Evaluation of nootropic and neuroprotective effects of low dose aspirin in rats

**DOI:** 10.4103/0976-500X.77079

**Published:** 2011

**Authors:** Arijit Ghosh, V. R. Dhumal, A. V. Tilak, Nina Das, Amarinder Singh, Abhijit A. Bondekar

**Affiliations:** *Department of Pharmacology, N.R.S. Medical College, Kolkata, India*; 1 *Department of Pharmacology, Modern Dental College, Indore, Madhya Pradesh, India*; 2 *Department of Pharmacology, Padmashree Dr. D.Y. Patil Medical College, Pimpri, Pune, India*

**Keywords:** Aspirin, serotonergic transmission, conditioned avoidance response, lithium induced head twitches

## Abstract

**Objective:**

To evaluate the nootropic and neuroprotective effects of aspirin in Sprague Dawley rats.

**Materials and Methods:**

Retention of conditioned avoidance response (CAR) and central 5-HT-mediated behavior (lithium-induced head twitches) were assessed using repeated electroconvulsive shock (ECS) in rats. Rats were divided into eight groups: control (pretreated with distilled water), scopolamine (0.5 mg/kg i.p.), ECS (150 V, 50 Hz sinusoidal with intensity of 210 mA for 0.5 s) pretreated, aspirin (6.75 mg/kg orally) pretreated, combined scopolamine and aspirin pretreated, ondansetron (0.36 mg/kg orally) pretreated, combined ECS and ondansetron pretreated and combined ECS and aspirin pretreated groups. Data was analyzed by the chi-square test and ANOVA.

**Results:**

Findings show that administration of single ECS daily for consecutive 8 days results in enhancement of 5-HT-mediated behavior (lithium-induced head twitches) and in disruption of the retention of CAR. Aspirin and ondansetron administration significantly increased the retention of conditioned avoidance response compared to control. Ondansetron and aspirin significantly prevented ECS-induced attenuation of the retention of conditioned avoidance response also. On the other hand, ondansetron and aspirin significantly retarded the ECS-induced enhancement of 5-HT-mediated behavior.

**Conclusion:**

Inhibition of the serotonergic transmission by aspirin is responsible for its nootropic and neuroprotective actions.

## INTRODUCTION

It is becoming increasingly clear that aspirin, previously thought of as an old anti-inflammatory workhorse, is now approaching the status of a wonder drug, as it is of benefit not only in inflammation but also in an increasing number of other conditions such as Alzheimer’s disease (AD).[1] There is preliminary evidence that aspirin decreases the risk and delays the onset of AD. Users of high dose aspirin had significantly lower prevalence of Alzheimer’s dementia and better cognitive function than non-users of aspirin. On the other hand, low dose aspirin users had numerically lower prevalence of Alzheimer’s dementia and better cognitive function than non-users of aspirin.[2]

Although AD once thought to result from a cholinergic deficit alone, researchers now believe that this view is simplistic. Studies using human neocortical tissue have shown that multiple neurotransmitters including dopamine, noradrenaline, serotonin and glutamate have shown to be decreased or dysregulated in AD.[3–5] An inhibitory effect of aspirin and other NSAIDs on the processes contributing to AD and cognitive decline seems plausible, even if convincing evidence from controlled trials is still lacking. The possibility that a well documented and non-expensive drug such as aspirin might function to maintain cognitive function and reduce the development of AD makes aspirin useful for testing, as an agent against cognitive decline.[1]

Behavioral models for studying memory shortage and recall, and its manipulation by pharmacological agents, do not represent the pathophysiology of AD. Thus for the induction and simulation of pathophysiology of underlying AD in experimental animals, amnesia is induced in animals. Various amnesia-inducing agents are available e.g. scopolamine, colchicines, etc.[6] Following completion of a course of electroconvulsive therapy (ECT), memory is impaired and retrograde amnesia is seen in human beings. These adverse effects are major factors limiting the use of ECT as the memory disturbances occur quite frequently.[7] Chronic administration of ECS for 8-10 days induces amnesia in animals by facilitating serotonergic transmission.[8] In the present study, electroconvulsive shock (ECS) was given to rats in the ways closely mimicking the administration of ECT to evaluate the nootropic and neuroprotective effects of aspirin in Sprague Dawley rats.

## MATERIALS AND METHODS

### Animals

Experimentally naive Sprague Dawley albino rats weighing between 150 and 200 g of either sex were used. The rats were maintained under standard conditions of temperature (25°C±5°C), relative humidity (55±10%) and a 12/12 h light/dark cycle. The rats were fed with commercial rat pellet diet manufactured by Pranav Agro Food, Pune and water *ad libitum*. The study was approved by the Institutional Animal Ethics Committee.

### Instruments, drugs and chemicals

Electro-convulsiometer and Cook’s pole climbing apparatus were purchased from ST1 Instruments Private Ltd and New Neeta Manufacturer, Pune, India respectively. Aspirin was purchased from Ranbaxy Laboratories Ltd, Gurgaon, India. Ondansetron was purchased from Sun Pharmaceutical Industries Ltd, Mumbai, India. Scopolamine was obtained from Sigma Aldrich, St Louis, USA. Lithium chloride (anhydrous) was purchased from Thomas Baker Ltd, Mumbai, India.

### Conditioned avoidance response

This model was used to study the neuroprotective and nootropic effects of aspirin. The rats were trained for conditioned avoidance response by using Cook’s pole climbing apparatus.[8,9] Each rat was allowed to acclimatize for 2 min and was then exposed to a buzzer noise. After 5 s of putting on the buzzer, mild electric shocks were given through the stainless steel grid floor. The magnitude of the voltage was adequate (10 V) to stimulate the rat to escape from the floor and climb the pole. As soon as the rat climbed the pole, both the buzzer and foot-shock button were switched off. At least 10 such trials were given to each rat at an interval of 1 min per day for 10 days. After about 10 days training schedule, most of the rats learned to climb the pole within 5 s of starting the buzzer to avoid the electric foot shocks. Rats, avoiding the foot shocks in all 10 out of 10 trials, were considered to have developed conditioned avoidance response for further experiments. All trained rats were divided randomly into eight groups and were used as follows:

**Table:** 

Group I (n=20):	Served as control
Group II (n=20):	Positive control (scopolamine 0.5 mg/kg i.p.)
Group III (n=18):	ECS (150 V, 50 Hz sinusoidal with intensity of 210 mA for 0.5 s through crocodile clip ear electrodes once daily for 8 days).
Group IV (n=20):	Aspirin 6.75 mg/kg per orally for 8 days.
Group V (n=20):	Scopolamine (0.5 mg/kg i.p.) +Aspirin (6.75 mg/kg per oral after 20 min of scopolamine injection for 8 days)
Group VI (n=20):	Ondansetron 0.36 mg/kg per orally for 8 c days.
Group VII (n=20):	ECS (150 V, 50 Hz sinusoidal with intensity of 210 mA for 0.5 s) and ondansetron (0.36 mg/kg per oral after 1h of ECS for 8 days)
Group VIII (n=18):	ECS (150 V, 50 Hz sinusoidal with intensity of 210 mA for 0.5 s) and aspirin (6.75 mg/kg per oral after 1 h of ECS for 8 days)

On day 9, all rats were tested to see if they had retained the conditioned avoidance response. After 2 min of acclimatization period, each rat was exposed to the buzzer for 5 s. Ten such trials were given at an interval of 1 min, without giving any foot shock. Rats, responding by climbing the pole when exposed to the buzzer noise, were considered to have retained the conditioned avoidance response.

### Lithium-induced head twitches (5-HT-mediated behavior)[10]

This model was used to study the interaction of aspirin with 5-HT. The number of head twitches induced by lithium (150 mg/kg; i.p.)was counted in intervals of 10 min, starting immediately from the time of injection up to a period of 90 min. The animals were divided into eight groups of six rats each as done in the previous model. Pretreatments in these eight groups were carried out in the same manner and, on day 9, evaluation was carried out.

### Statistical analysis

The result of the lithium-induced head twitches was analyzed by ANOVA followed by Turkey’s honestly significant difference (HSD) test. The result of the retention of CAR was analyzed by the chi-square test. P<0.05 was consider as significant.

## RESULTS

### Conditioned avoidance response

**Figure 1:**
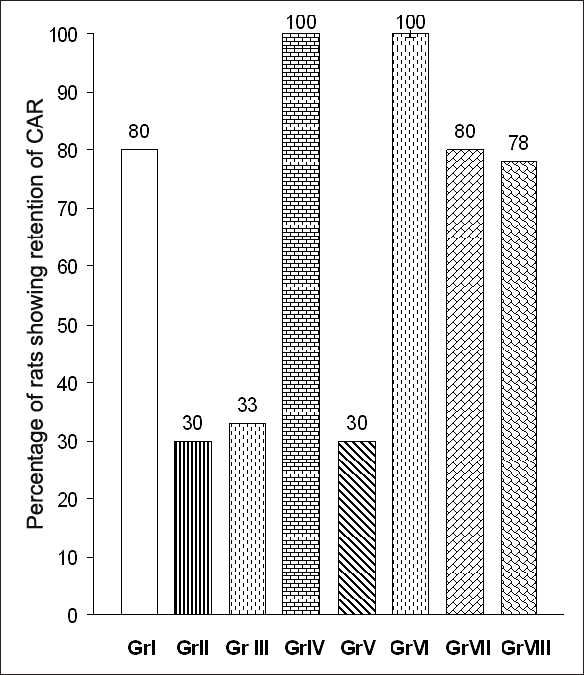
Percentage of rats showing retention of conditioned avoidance response. Gr II, III, IV, VI Vs Gr I (*P*<0.001)

The percentage of rats showing retention of conditioned avoidance response (CAR) was calculated in each group. The result is shown in Figure 1. In group I, 80% rats showed retention of CAR. In group II 30% rats and in group III 33% rats showed retention of CAR, and these decreases were statistically significant (*P*<0.001) compared to group I. In groups IV and VI 100% rats showed retention of CAR, and these increases were statistically significant compared to group I (*P*<0.001). In comparison to group III, groups VII and VIII showed an increase in the percentage of rats showing retention of CAR and this increase was statistically significant (*P*<0.001).

### Lithium-induced head twitches

**Figure 2:**
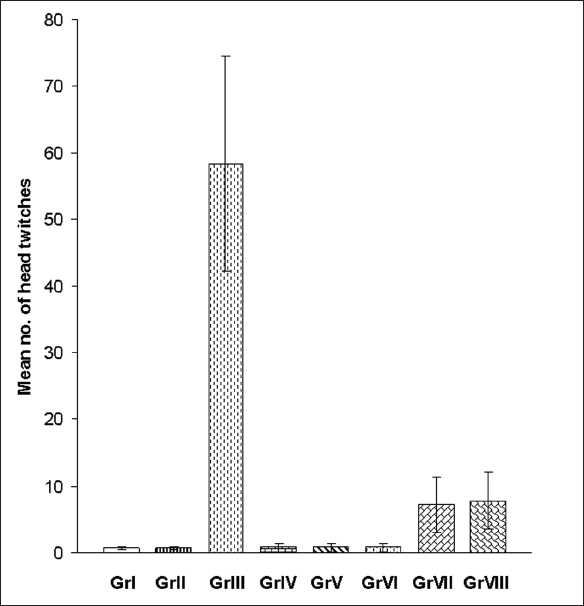
Number of lithium-induced head twitches during 31-40 min interval. Gr VII, VIII Vs Gr III (*P*<0.001)

The number of head twitches induced by injecting lithium chloride was counted every 10 min, starting immediately from the time of injection up to a period of 90 min. The results of this study are shown in Figure 2. The maximum number of head twitches in different groups was seen between 31 and 40 min. So, head twitches at this interval were compared in different groups. The number of head twitches was 0.67± 0.21 (mean ±S.E.M.) in group I. It increased to 58.33±16.17 in group III, and this increase was statistically significant as compared to group I (*P*<0.001). In comparison to group III, number of head twitches decreased in groups VII and VIII, and this decrease was statistically significant (*P*<0.001).

## DISCUSSION

The present study was planned to see if aspirin produced its action by significantly influencing serotonergic systems in the CNS, that might prove to be of importance in treating patients, not only of Alzheimer’s disease but of other diseases of CNS also. Recent laboratory findings suggest that aspirin play role in neuroprotection.[11] One case has been reported of a woman whose electroencephalogram (EEG) abnormalities reversed and whose psychological symptoms regressed after treatment with low doses of aspirin. This is the first description of a disappearance of EEG abnormalities with low doses of aspirin. The neuroprotective effect of aspirin can be of extreme importance in metabolic neuronal dysfunction, where both clinical symptoms and EEG genetic anomalies could be reversed. The neurological dysfunction is probably due to disequilibrium of concentration of the neurotransmitters caused by sub-clinical hypoxic changes in the cortex.[12] Aspirin seems to restore the equilibrium of concentration of the neurotransmitters.

Our study shows that administration of single ECS daily for consecutive 8 days results in enhancement of 5-HT-mediated behavior (lithium-induced head twitches) and in disruption of the retention of CAR. Aspirin and ondansetron administration significantly increased the retention of conditioned avoidance response compared to control. Ondansetron and aspirin significantly prevented ECS-induced attenuation of the retention of conditioned avoidance response also. On the other hand, ondansetron and aspirin significantly retarded the ECS-induced enhancement of 5-HT-mediated behavior.

Head twitches in rats can be used to study 5-HT neuronal activity. Several drugs induce these in rats, i.e. 5- hydroxytryptophan (5-HTP) by increasing free concentration of 5-HT at its receptor site or 5-methoxytryptamine by directly mimicking endogenous 5-HT at its receptor sites. Head twitches also occur in rats treated with lithium chloride and constitutes an useful animal model for quantifying 5-HT activity in the brain and screening of potential antagonists of 5-HT receptors.[10] Fenfluramine, a serotonin reuptake inhibitor and releasing agent, which effectively increases serotonin levels appeared to impair the working memory in human subjects. Evidences suggest that serotonergic transmission inhibits the working memory performances and it is also suggested that blocking serotonergic transmission in brain is a possible mechanism to enhance the working memory performances.[8,13] The result of lithium-induced head twitches is in agreement with the fact that blocking serotonergic transmission in the brain is a mechanism to enhance the retention of conditioned avoidance response. Thus, the results in the present study suggest the role of serotonergic system in nootropic and neuroprotective effects of low dose aspirin.
